# Characteristics of Three Thioredoxin Genes and Their Role in Chilling Tolerance of Harvested Banana Fruit

**DOI:** 10.3390/ijms17091526

**Published:** 2016-09-09

**Authors:** Fuwang Wu, Qing Li, Huiling Yan, Dandan Zhang, Guoxiang Jiang, Yueming Jiang, Xuewu Duan

**Affiliations:** Key Laboratory of Plant Resources Conservation and Sustainable Utilization/Guangdong Provincial Key Laboratory of Applied Botany, South China Botanical Garden, Chinese Academy of Sciences, Guangzhou 510650, China; 2004370322@163.com (F.W.); liqing209@126.com (Q.L.); hlingyan@scbg.ac.cn (H.Y.); ddzhang@scbg.ac.cn (D.Z.); xiangzi13145206@163.com (G.J.); ymjiang@scbg.ac.cn (Y.J.)

**Keywords:** banana fruit, thioredoxin, chilling injury, ethylene

## Abstract

Thioredoxins (Trxs) are small proteins with a conserved redox active site WCGPC and are involved in a wide range of cellular redox processes. However, little information on the role of Trx in regulating low-temperature stress of harvested fruit is available. In this study, three full-length *Trx* cDNAs, designated *MaTrx6*, *MaTrx9* and *MaTrx12*, were cloned from banana (*Musa acuminata*) fruit. Phylogenetic analysis and protein sequence alignments showed that MaTrx6 was grouped to h2 type with a typical active site of WCGPC, whereas MaTrx9 and MaTrx12 were assigned to atypical cys his-rich Trxs (ACHT) and h3 type with atypical active sites of GCAGC and WCSPC, respectively. Subcellular localization indicated that MaTrx6 and MaTrx12 were located in the plasma membrane and cytoplasm, respectively, whereas MaTrx9 showed a dual cytoplasmic and chloroplast localization. Application of ethylene induced chilling tolerance of harvested banana fruit, whereas 1-MCP, an inhibitor of ethylene perception, aggravated the development of chilling injury. RT-qPCR analysis showed that expression of *MaTrx12* was up-regulated and down-regulated in ethylene- and 1-MCP-treated banana fruit at low temperature, respectively. Furthermore, heterologous expression of MaTrx12 in cytoplasmic Trx-deficient *Saccharomyces cerevisiae* strain increased the viability of the strain under H_2_O_2_. These results suggest that MaTrx12 plays an important role in the chilling tolerance of harvested banana fruit, possibly by regulating redox homeostasis.

## 1. Introduction

Bananas (*Musa acuminata*) are a major staple food and export product in many countries, with an annual output of 102 million tons worldwide [1]. Bananas are a climacteric fruit and undergo a rapid ripening process after harvest, leading to a short shelf life. Harvested banana fruit is sensitive to ethylene. Exposure of banana fruit to ethylene as low as 0.1 ppm is sufficient to initiate ripening [2]. Conversely, a very low level of 1-MCP, an inhibitor of ethylene perception, inhibits banana fruit ripening. Silser and Serek reported that 0.7 ppb 1-MCP delays banana fruit ripening for 12 days at 24 °C [3]. Jiang et al. reported that exposure for 12 h at 20 °C to just 50 ppb essentially eliminates ethylene-stimulated ripening effects [4]. Despite the efficiency of 1-MCP in inhibiting banana fruit ripening, in practice, it is difficult for 1-MCP to be used in postharvest handling of banana fruit because 1-MCP treatment usually results in abnormal ripening.

Low temperature storage is effective in prolonging storage and shelf life of banana fruit. However, chilling injury symptoms occur when the storage temperature is less than 12 °C, manifesting as peel browning and failure to ripen [5,6]. Ethylene plays a role in cold stress response in harvested fruit. Ethylene has been reported to accelerate chilling injury symptoms of plum [7] and avocado [8], but alleviate the development of chilling injury in nectarine [9]. It has also been shown that 1-MCP, an inhibitor of ethylene perception, aggravates chilling injury severity in bananas [6] and peaches [10]. Nevertheless, chilling injury symptoms of apples [11], avocados [8], plums [7], okra [12] and loquat [13] are obviously relieved by 1-MCP. Therefore, different species of fruits show different responses to ethylene when subjected to low temperature stress. Our preliminary study has shown that ethylene pretreatment alleviated the development of chilling injury in harvest banana fruit. However, the underlying mechanism involved in induced chilling tolerance by ethylene in fruit remains largely unknown.

Thioredoxins (Trxs) are a kind of small and widely distributed protein with a conserved active site motif (CGPC), which controls the redox status of target proteins through thiol-disulfide exchange reactions [14]. Mammalian cells possess only two Trxs isoforms, the cytoplasmic Trx1 and the mitochondrial Trx2, which are involved in transferring electrons to peroxiredoxins and methionine sulfoxide reductases, regulating the activities of some redox-sensitive transcription factors, and signaling of apoptosis [15,16,17,18,19]. In plant, Trxs are encoded by a multigene family [20]. Trxs plays a fundamental role in a number of cellular processes in plants, including seed germination, carbon assimilation, lipid metabolism, hormone metabolism, redox signaling, and stress response [14,21,22,23,24,25,26]. Trxs are implicated in the oxidative stress responses in plants by (1) repairing oxidative proteins (such as iron-sulfur protein and DNA damage repair related proteins) [23]; (2) activating the activity of the protecting enzymes in the antioxidant system [27]; and (3) acting as regulators of scavenging mechanisms or signaling pathways in the antioxidant network [25,28,29]. Currently, most research on Trxs in relation to oxidative stress response is focused on the model plant *Arabidopsis* [25,30,31]. The involvement of Trx in cold tolerance in rice [32] and potatoes [33] has also been reported. However, there are differences in response to low temperature between fruit unattached from the tree and the plant. Unfortunately, little information on the role of Trx in chilling tolerance of harvested fruits is available.

In this study, three full-length *MaTrx* cDNAs were cloned from banana fruit and their structure characteristics and subcellular localization were analyzed. In addition, ethylene and 1-MCP pretreatments were applied to evaluate the expression of three *MaTrx* genes in bananas subjected to various degrees of chilling injury. Furthermore, heterologous complementation experiments with cytoplasm Trx-deficient *Saccharomyces cerevisiae* strain were performed to analyze the role of these three MaTrxs in oxidative stress tolerance. The results will help to further understand the mechanism underlying occurrence and regulation of chilling injury in harvested banana fruit.

## 2. Results

### 2.1. Cloning of MaTrx6, MaTrx9 and MaTrx12 Genes from Banana Fruit

Conserved fragments of three banana fruit *Trx* genes, *MaTrx6*, *MaTrx9* and *MaTrx12* were isolated from peel tissue using the degenerated primers by RT-PCR, and their full length cDNAs were obtained using the RACE strategy, with the lengths of 659, 1133 and 1044 bp, respectively. *MaTrx6*, *MaTrx9* and *MaTrx12* genes were predicted to encode the proteins with 127, 272 and 144 amino acids, respectively.

### 2.2. Sequence and Phylogenetic Analysis of Banana Fruit Trx Genes

**Figure 1:**
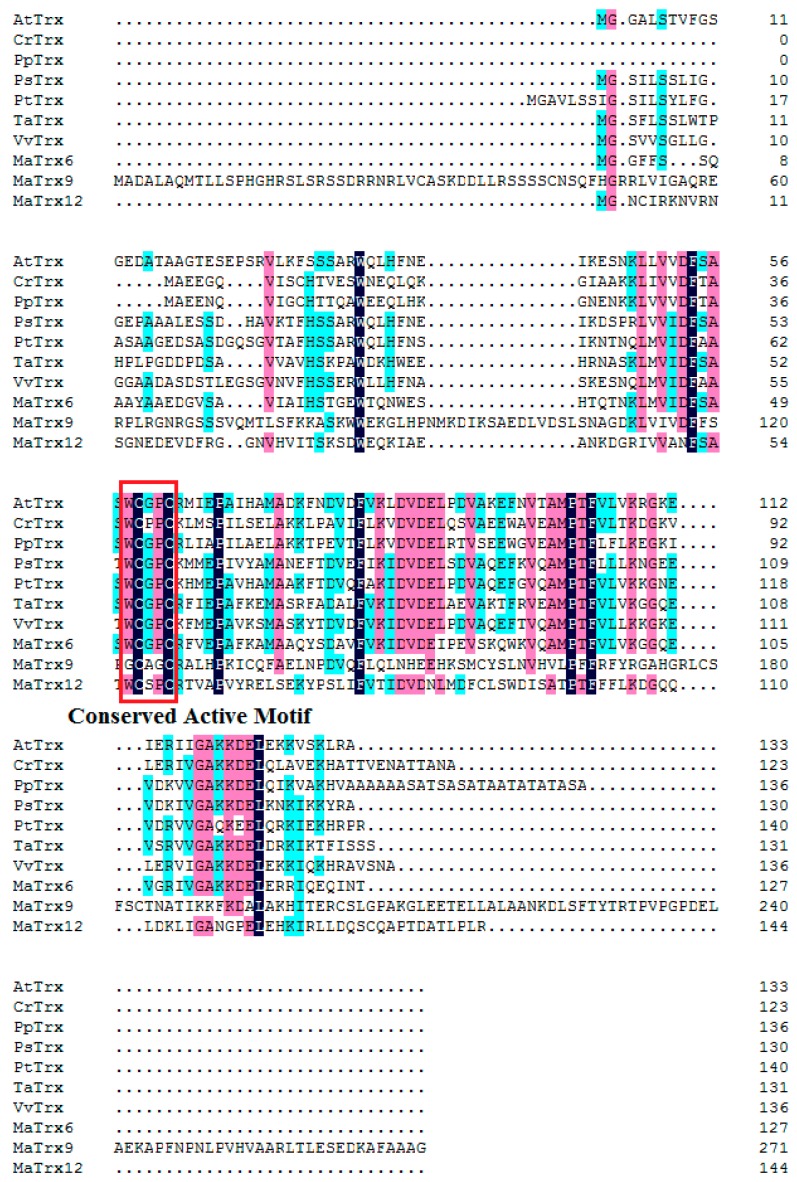
Homology comparison of amino acid sequence alignment of banana (*Musa acuminata*) Trxs with other plants. The selected sequences were Trx from *Citrus reticulata* (AAP33009.1), *Prunus persica* (AAL26915.1), *Pisum sativum* (AAO12855.1), *Populus tomentosa* (XP_002324032.2), *Arabidopsis thaliana* (AAC49353.1), *Triticum aestivum* (ACH61777.1) and *Vitis vinifera* (XP002282318.1) by DNAMAN V8. The conserved active motif was boxed.

**Figure 2:**
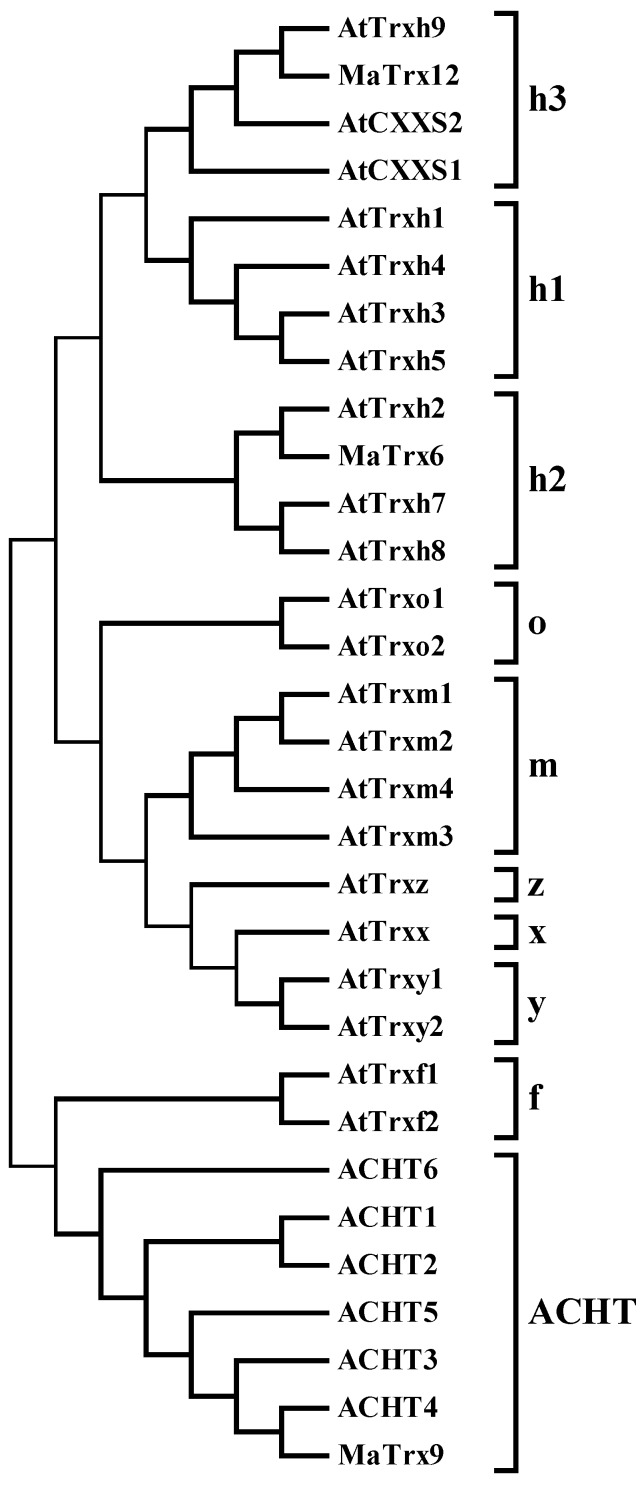
Phylogenetic tree of MaTrx6, MaTrx9 and MaTrx12 grouped with different types of *Arabidopsis thaliana* Trxs. Members of different subgroup of Trx were clustered, and the AGI numbers corresponding to each protein were AT3G51030 (h1), AT5G42980 (h3), AT1G19730 (h4), AT1G45145 (h5), AT5G39950 (h2), AT1G59730 (h7), AT1G69880 (h8), AT3G08710 (h9), AT1G11530 (CXXS1), AT2G40790 (CXXS2), AT2G35010 (o1), AT1G31020 (o2), AT3G02730 (f1), AT5G16400 (f2), AT1G03680 (m1), AT4G03520 (m2), AT2G15570 (m3), AT3G15360 (m4), AT1G50320 (x), AT1G76760 (y1), AT1G43560 (y2), AT3G06730 (z), AT1G08570 (ACHT1), AT4G29670 (ACHT2), AT5G61440 (ACHT3), AT2G33270 (ACHT4), AT4G26160 (ACHT5), AT1G07700 (ACHT6).

Sequence analysis showed that three MaTrxs had high similarity with other published Trx proteins from higher plants (Figure 1). MaTrx6 had a 62% similarity to TaTrx (ACH61777.1), MaTrx9 a 71% similarity to VvTrx (XP002282318.1), and MaTrx12 a 69% similarity to PpTrx (AAL26915.1). In addition, the conserved active site motif sequences differed among them. MaTrx6 had a typical WCGPC active site, whereas MaTrx9 and MaTrx12 exhibited atypical active sites, GCAGC and WCSPC, respectively. Moreover, a phylogenetic tree was produced using the neighbor-joining (NJ) method with MEGA 5 software. According to *Arabidopsis thaliana* classification, MaTrx6, MaTrx9 and MaTrx12 were grouped into h2 type, ACHT type and h3 type (Figure 2).

### 2.3. Subcellular Localization of MaTrx6, MaTrx9 and MaTrx12

**Figure 3:**
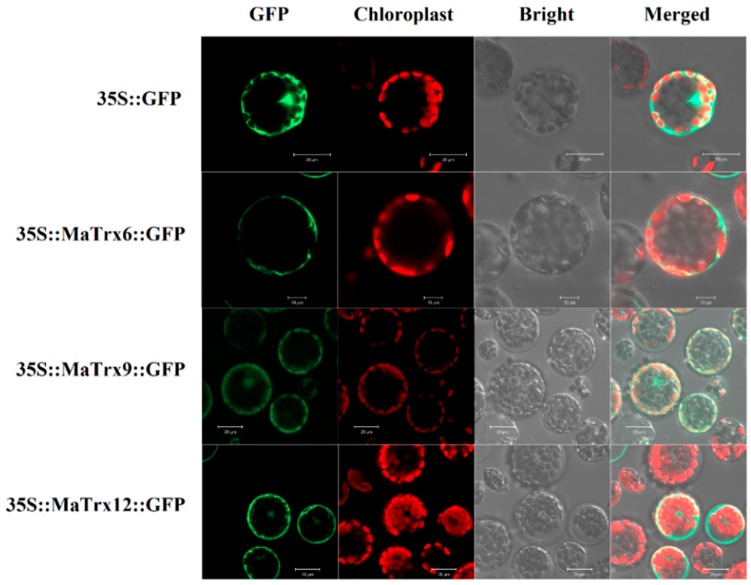
Subcellar localization of MaTrx6, MaTrx9 and MaTrx12 in *Arabidopsis* protoplast. The green fluorescene from three proteins merged with bright field and red fluorescene from the chloroplast using confocal microscopy. GFP: green fluorescent protein. In 35::GFP, 35S::MaTrx9::GFP and 35S::MaTrx12::GFP, the scale bar = 10 μm. In 35S::MaTrx6::GFP, the scale bar = 20 μm.

To investigate the subcellular localization of MaTrx6, MaTrx9 and MaTrx12 proteins in vivo, we cloned their ORFs into a transformation vector (pUC18-GFP) fused with a GFP reporter gene under the control of the CaMV-35S promoter. Expressions of MaTrx6, MaTrx9 and MaTrx12-GFP proteins in *A*. *thaliana* protoplasts by polyethylene glycol mediated transfection were expected to indicate their proper subcellular localizations in a native setting (Figure 3). The GFP control showed ubiquitous distribution throughout the whole cell. MaTrx6 protein was localized to the plasma membrane, whereas MaTrx9 protein showed a dual cytoplasmic and chloroplast localization in the cells. The green fluorescence of MaTrx12 protein was clearly detected in the cytoplasm.

### 2.4. Effect of Ethylene and 1-MCP Pretreatments on the Development of Chilling Injury in Harvested Banana Fruit

**Figure 4:**
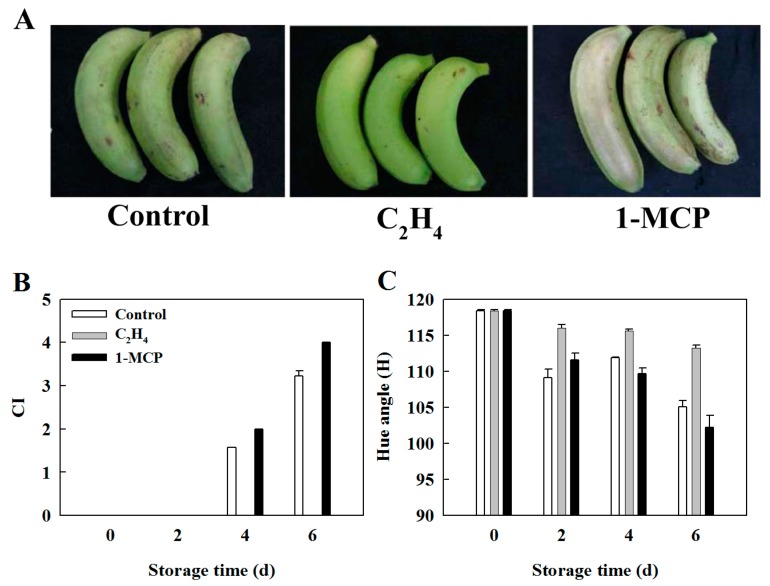
Visual appearance and physiological characterization of banana fruit stored at 6 °C. Visual appearance (**A**) of banana fruit treated with ethylene and 1-MCP for 12 h after 6 days of storage at 6 °C. Effect of ethylene and 1-MCP pretreatments on chilling injury (**B**) and color parameter (**C**) of banana fruit stored at 6 °C. The hue angle of 120 means green color while 90 means yellow color.

At the early stage of chilling injury, the banana fruit skin slightly darkened, lost glossiness and developed some depression dots. As chilling injury progressed, the banana peel experienced browning, associated with lignification of vascular bundle and water soaking (Figure 4A). Compared with control fruit, 1-MCP pretreatment accelerated the development of chilling injury in harvested banana fruit. After 6 days of storage, the chilling injury index was approximately 4.0. However, fruit treated with ethylene showed no chilling injury symptom 6 days after storage at 6 °C (Figure 4B), suggesting that ethylene plays a role in chilling tolerance in harvested banana fruit. Moreover, peel hue angle decreased with increased chilling injury severity. Consistent with the development of chilling injury, higher and lower hue values were observed in ethylene- and 1-MCP-treated fruit, respectively, compared with control fruit (Figure 4C).

### 2.5. Expression of MaTrx6, MaTrx9 and MaTrx12 Genes in Ethylene- and 1-MCP-Treated Banana Fruit in Response to Low Temperature Stress

**Figure 5:**
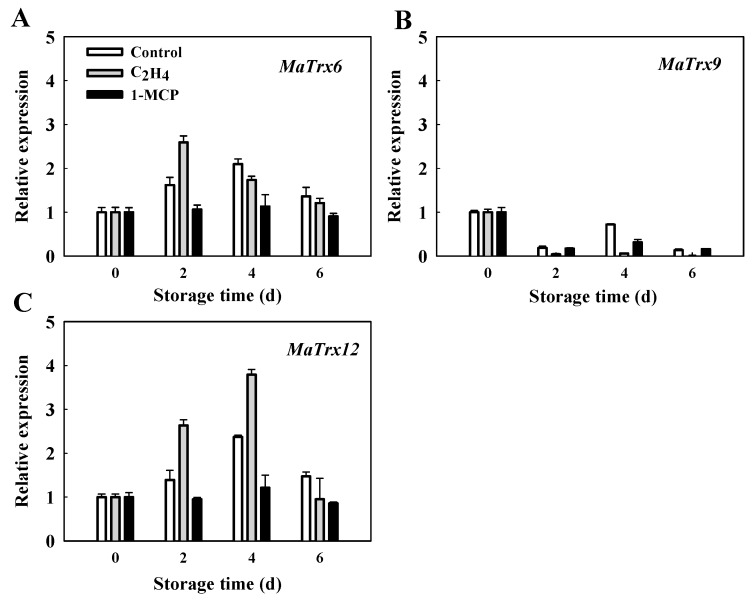
Effect of ethylene and 1-MCP treatments on expression of *MaTrx6* (**A**), *MaTrx9* (**B**) and *MaTrx12* (**C**) genes in the peel of banana fruit stored at 6 °C. *MaACT1* was used as internal control. The expression of the genes at 0 day was set to 1.0. The data were the means of three independent biological replicates with similar results.

As shown in Figure 5, low temperature stress induced the significant expression of *MaTrx6* and *MaTrx12* genes in control fruit. Compared with control fruit, expression of *MaTrx6* gene was up-regulated at 2 days and that of *MaTrx12* was up-regulated at 2 and 4 days by ethylene pretreatment. After 1-MCP treatment, expression of *MaTrx6* and *MaTrx12* genes were almost constant during storage at low temperature. Different from *MaTrx6* and *MaTrx12*, expression of *MaTrx9* gene was inhibited when the fruit was transferred to low temperature. These results indicated that *MaTrx12* possibly played a more important role in the chilling tolerance of harvested banana fruit.

### 2.6. Effect of MaTrx6, MaTrx9 and MaTrx12 Heterologous Expression on the Growth of a Cytoplasmic Trx-Deficient Yeast Strain under Hydrogen Peroxide Treatment

**Figure 6:**
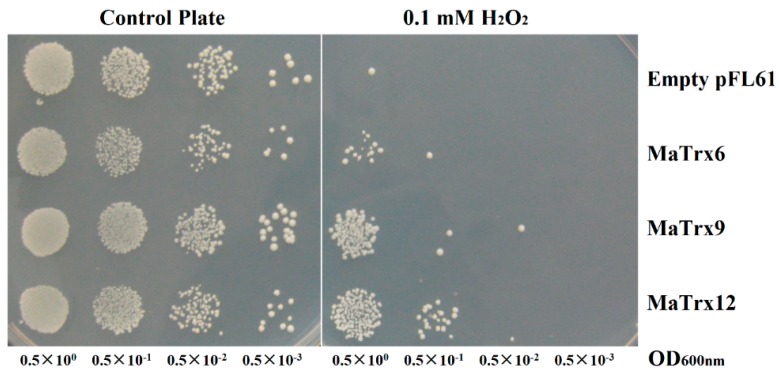
Functional complementation of *Trx1*Δ*Trx2*Δ yeast cells by heterologous MaTrx6, MaTrx9 and MaTrx12, respectively. Yeast expressing MaTrxs were grown to a density of 10^7^ cells·mL^−1^. 30 μL of serial dilutions (OD_600nm_ = 0.5 × 10^0^, 0.5 × 10^−1^, 0.5 × 10^−2^, 0.5 × 10^−3^) were plated on SC-Ura medium without (control plate) or containing 0.1 mM hydrogen peroxide, and the empty pFL61 vector was used as control.

To analyze the possible involvement of MaTrxs in protecting against oxidative stress, the mature forms of heterologous MaTrx6, MaTrx9 and MaTrx12 were transformed into *Saccharomyces cerevisiae* EMY63 strain which is deficient for cytoplasm Trxs and sensitive to oxidative stress. Transformed positive clones were plated on SC-Ura medium supplemented with 0.1 mM H_2_O_2_, and their growth capacities were monitored after 72–96 h at 30 °C. The results showed that there was no obvious difference in growth from the control, and MaTrx6, MaTrx9 and MaTrx12 overexpressed *Saccharomyces cerevisiae* strains in the medium without H_2_O_2_. EMY63 strain exhibited almost no growth when subjected to H_2_O_2_ stress. However, MaTrx9- and MaTrx12-complemented EMY63 strains grew well in the medium containing 0.1 mM H_2_O_2_ (Figure 6).

## 3. Discussion

Low temperature storage is very effective in extending storage and shelf life of harvested horticultural crops [34]. However, tropical and subtropical fruits and vegetables are susceptible to chilling injury when stored at less than 12 °C. As shown in Figure 4, severe chilling injury symptoms have developed in harvested banana fruit after 6 days of storage at 6 °C. Reactive oxygen species (ROS) accumulation is considered to be one of the primary causes of chilling injury [35]. Wang et al. [36] and Huang et al. [37] reported the accumulation of ROS in harvested banana fruit as chilling injury progressed. ROS are highly reactive and usually cause oxidative damage of macromolecules, especially proteins, leading to loss of function [19]. It is well known that the maintenance of protein quality is important for organisms to cope with stress conditions. Heat shock proteins are a stress-responsive family of proteins and involved in abiotic tolerance in plants by protecting proteins from denaturation and dysfunction, or functioning as a chaperone. Numerous studies have shown that Hsps play an important role in chilling tolerance of harvested fruits [38]. In addition to Hsps, few proteins associated with protein quality control in harvested fruits were reported.

Thioredoxins (Trxs) are an important kind of protein related to protein modification. Trxs are characterized by a conserved active site WCGPC, which is able to reduce the disulfide bridges of target proteins [14]. Animals contain only two types of Trxs, while Trxs are encoded by a multigene family in higher plants [14]. In *Arabidopsis*, at least forty Trxs have been reported and classified into 15 subfamilies, Trxh (h1, h2 and h3), Trxo, Trxm, Trxf, Trxx, Trxy, Trxz, CDSP32, WCRKC, ACHT, TDX, Clot, Nrx, Picot and Atypical chloroplastic Trx [20]. In the present study, three full-length *Trx* cDNAs, designated *MaTrx6*, *MaTrx9* and *MaTrx12*, were first obtained from banana fruit (Figure 1). According to the classification in *Arabidopsis*, MaTrx6, MaTrx9 and MaTrx12 belonged to h2, ACHT and h3 type, respectively (Figure 2). The functions of different Trxs are correlated with their structure and subcellular localization [20]. In the present study, MaTrx6 contained a typical active site of WCGPC, whereas the catalytic active centers of MaTrx9 and MaTrx12 mutated to GCAGC and WCSPC, respectively (Figure 1). Collet and Messens proposed that, in addition to the two cysteine residues, other conserved residues are important for activity, redox and thermodynamic properties of the proteins [14]. Gelhaye et al. reported that the mutant from WCGPC to WCPPC greatly alters the conformation of PtTrx h3 [39]. In *E. coli*, the replacement of the proline (P) by serine (S) or threonine (T) results in remarkable decrease in the stability and reduction activity of Trx [40]. It is suggested that the changes in the structures of MaTrx9 and MaTrx12 might affect their stability and activity in vivo.

In addition, subcellular localization showed that MaTrx6 and MaTrx12 were mainly located in plasma membrane and cytoplasm, respectively (Figure 3), which was in accordance with other plant Trxh [20]. MaTrx9, belonging to ACHT type, was distributed both in chloroplast and cytoplasm (Figure 3). Many light response elements such as AAAC-motif, ACE and LAMP-element were present in the *MaTrx9* promoter (data not shown), which possibly were related to its chloroplast localization. Moreover, there was a chloroplast transit peptide (30 AA) predicted in the N-terminus of MaTrx9 (data not shown). ACHT was reported to be a chloroplast atypical thioredoxin rich in cysteine and histidine residues, functioning in modulating the redox state and activity of chloroplast proteins with regulatory disulfides [41]. The differences in active motif sequence and subcellular localization suggest that MaTrx6, MaTrx9 and MaTrx12 might play various roles in growth, development and stress responses in banana.

Thioredoxins play an important role in tolerance to oxidative stress in plant by reducing the disulfide bond formed from oxidation of cysteine residues by ROS [29]. There is mounting evidence that oxidative stresses induce *Trx* gene expression [42]. Shankar et al. compared the transcirptome profiles in different rice cultivars under drought stress and showed that the transcripts encoding Trx are un-regulated in drought-tolerant cultivar [43]. Xie et al. found that a number of *Trxs* are up-regulated in drought-treated tobacco leaves by iTRAQ-based quantitative proteomic analysis and proposed that redox-induced posttranslational modifications play an important role in modulating protein activity in response to drought stress [44]. Our results showed that the expression of *MaTrx6* and *MaTrx12* genes were up-regulated in banana peel by low temperature stress (Figure 5), suggesting that the oxidation of protein cysteine residues occurred on a large scale. The increased expression of *MaTrx6* and *MaTrx12* genes possibly were beneficial for reducing the disulfide bonds of oxidized proteins. However, the expression of *MaTrx9* gene was inhibited by low temperature stress. It can be explained that various Trxs might play differential roles in development or abiotic stress in banana fruit.

To gain better insight into the role of MaTrx in chilling tolerance of harvested banana fruit, two ethylene-related pretreatments were applied. The response of different species of fruits to ethylene under low temperature stress varies. Ethylene accelerates chilling injury-related disorders in some fruits, but alleviates the development of chilling injury in other fruits [7,8,9]. In this study, the fruit treated with ethylene showed no chilling injury symptom, whereas 1-MCP, an inhibitor of ethylene perception, aggravated the development of chilling injury (Figure 4), suggesting that ethylene plays a positive regulatory role in chilling tolerance of harvested banana fruit. There are quite a few reports that cold tolerance in some climacteric fruits is related to the maturity at harvest. Qian et al. reported that cucumber fruit at the earlier developmental are more susceptible to chilling injury, which is associated with increased oxidative stress [45]. Similarly, stronger resistance to chilling injury in yellow mature mango is due to higher antioxidant capacity, compared with green mature fruit [46]. However, ethylene pretreatment did not prompt banana fruit ripening during storage at 7 °C [47]. It appears that the induced cold tolerance by ethylene has no relation to fruit maturity. Furthermore, expression of the *MaTrx12* gene was significantly up-regulated by exogenous ethylene but down-regulated by 1-MCP treatment (Figure 5), consistent with the chilling tolerance induced by ethylene. Accumulating evidence indicates that Trxs are involved in cold tolerance in growth and development in plants. Rorat et al. reported that CDSP32, a plastidic thioredoxin, is highly expressed at low temperatures in the cold-tolerant *Solanum* species [33]. In rice, OsTrx23 is a cold-induced thioredoxin h and negatively regulates redox status of MAPKs [32]. Moon et al. further reported that overexpression of *AtNTRC*, containing a C-terminal thioredoxin domain, confers freezing and cold tolerance in *Arabidopsis thaliana* [48]. Therefore, it is suggested that MaTrx12 mediates ethylene-induced chilling tolerance in harvested banana fruit.

Considering the importance of ROS accumulation in the development of chilling injury, we further evaluated the role of MaTrx6, MaTrx9 and MaTrx12 in oxidative stress tolerance using a heterologous complementation method. EMY63, a cytoplasmic Trx-deficient *Saccharomyces cerevisiae* strain, is very sensitive to oxidative stress [49] and is a powerful tool to verify antioxidant function of plant Trx [50,51]. Our results showed that MaTrx12 complemented Trx-deficient EMY63 for hydrogen peroxide tolerance, and exhibited more activity in peroxide detoxification than MaTrx6 and MaTrx9 (Figure 6). Similarly, *Trxo1* overexpression enhances the viability of *Nicotiana tabacum* BY-2 cells under H_2_O_2_ treatment [52]. The difference in oxidative stress tolerance was also observed in Arabidopsis [53]. Our results suggest that MaTrx12 plays an important role in chilling tolerance of harvested banana fruit, possibly by regulating redox homeostasis of proteins-related to ROS scavenging. Surprisingly, although MaTrx9 also complemented EMY63 cells under H_2_O_2_, expression of *MaTrx9* was repressed in banana fruit treated with ethylene or 1-MCP treatment. In plant, Trxs are encoded by a multigene family. These genes could play different roles in different physiological processes or stress responses, i.e., certain *Trx* genes play a role in certain physiological processes, but not in others. In a preliminary study, we analyzed the expression of *MaTrx9* in different banana tissues, including peel, ovary, leaf and stem, and found that *MaTrx9* expression in the ovary was enormously higher than that in peel, leaf and stem (data not shown), suggesting that MaTrx9 possibly plays a role in fruit development.

## 4. Materials and Methods

### 4.1. Plant Materials and Treatments

Banana fruit (*Musa acuminata* L. AAA group, cv. Brazilian) were harvested at the mature green stage from an orchard at Huizhou City, Guangdong Province, China. The fruit were separated into fingers, washed with tap water, dipped in 0.05% Sportak^®^ (Prochloraz, Bayer, Leverkusen, Germany) solution for 3 min, and then dried in the air. Fruit with uniform size, shape and color were selected and randomly divided into three groups. Each group included three subgroups with 24 fruits each subgroup. One group was fumigated with 500 ppm C_2_H_4_ in sealed box for 12 h. The second group was treated with 5 ppm 1-MCP instead of ethylene. The control group was sealed in the same volume of box for 12 h. After treatments, the fruit were packaged with 0.03 mm-thick PVC bags to prevent water loss and stored at 6 °C and 85% RH for 6 day. Peel tissues for assessment were taken at 2-day intervals during storage, frozen in liquid nitrogen and stored at −80 °C for further analysis.

### 4.2. Measurement of Chilling Injury (CI) Index

Chilling injury (CI) was evaluated according to peel browning extent which was divided into five scales [54]: 0, no chilling injury; 1, slight browning; 2, less than 50% area showing browning; 3, 50%–75% area showing browning; 4, severe browning. CI index was calculated using the following formula:

CI index = ∑ (CI scale) × (number of each scale)/total number of fruit

### 4.3. Color Measurement

Fruit color were measured using a Chroma meter (Konica Minolta, CR-400, Tokyo, Japan) according to Mcguire et al. [55].

### 4.4. RNA Isolation and cDNA Synthesis

Total RNA from peel tissues was prepared according to the hot borate method [56], and digested with DNase I to remove the potentially contaminating DNA using an RNase-free kit (TianGen, Beijing, China). The DNA-free total RNA was then used as template for reverse transcription-PCR (RT-PCR).The Prime ScriptTM RT reagent Kit (Takara, Dalian, China) was used for synthesizing the first strand of cDNA according to the manufacturer’s instructions.

### 4.5. Amplification of MaTrx Full Length cDNAs

To isolate *MaTrx* cDNA, two synthetic degenerate oligonucleotide primers were designed according to the conserved peptide sequence: DFSATWCGP and VDFIKIDVDE, which were used as upstream primers to clone the 3’-end of cDNA with 3’race outer primer and 3’race inner primer of the 3’-Full RACE kit (Takara, Dalian, China). Then, 5’-rapid amplification of cDNA end (RACE)-PCR was performed using 5’-Full RACE kit (Takara, Dalian, China) according to the manufacturer’s protocol. The DNA sequences were confirmed by sequencing. The sequences of the designed primers used for PCR amplification and RACE are shown in Table 1.

### 4.6. Sequence and Phylogenetic Analysis

Identification of nucleotide sequences was established using the NCBI Blast program (http://www.ncbi.nlm.nih.gov/BLAST). Open reading frame and protein prediction were conducted using ExPaSy program (http://www.expasy.org). Alignment and comparison of sequences were performed using the DNAMAN V8 software (Lynnon Biosoft, Los Angeles, CA, USA). A phylogenetic tree was constructed using the neighbor-joining method in the MEGA 5 program.

### 4.7. Real-Time Quantitative PCR (RT-qPCR) Analysis

Total RNA were extracted and purified as above-mentioned method. Synthesis of first strand cDNA was performed with a Prime Script™ RT Master Mix kit (Takara, Dalian, China). MaACT1 was selected as reference gene according to Chen et al. [57]. The primers are shown in Table 1. The 20 μL reaction system of RT-qPCR included 10 μL SYBR^®^ Premix Ex TaqTM (2×), 0.4 μL Forward primer (10 μM), 0.4 μL Reverse primer (10 µM), 4 μL cDNA of each sample (5 ng/μL), 0.4 μL ROX Reference Dye II (50×), 4.8 μL RNase-free H_2_O. RT-qPCR program initially started with 95 °C for 3 min followed by 35 cycles of 95 °C for 5 s, 55 °C for 10 s and 72 °C for 30 s. The relative expression levels were the means of three independent biological replicates.

### 4.8. Subcellular Localization of MaTrxs

The ORF sequence of MaTrx without the stop codon was amplified by PCR and sub-cloned into the SalI or MluI site of a pUC18-GFP vector with a Cauliflower mosaic virus (CaMV) 35S promoter (the primers were shown in Table 1), in frame with the green fluorescent protein (GFP) sequence. The resulting constructs were introduced into *Arabidopsis* protoplasts by polyethylene glycol (PEG) mediated transfection, which were from the leaf of *Arabidopsis thaliana* plant grown under long-day conditions (16 h light/8 h dark) at 22 °C for 4 weeks. Following incubation under a low light intensity for 16 h, the cells were imaged with a ZEISS-510Meta con-focal spectral microscope imaging system (Leica, Solms, Germany) in the wavelength of 488 nm for the GFP fluorescence and 543 nm for Chloroplast auto-fluorescence. All transient expression assays were repeated at least three times.

### 4.9. Heterologous Complementation Analysis

Complementation experiments were performed with *Saccharomyces cerevisiae* strain EMY63 [49] according to previous reports [50]. Banana Trx expression in yeast cells was carried out with the constitutive pFL61 vector [58]. The primers used for the cloning of Trxs into pFL61 were shown in Table 1. All constructs were introduced into EMY63 strain cells by the lithium acetate method [59], and the positive clone strains were then verified by PCR using FLDR/FLGA pairs of primer. When the yeast cultures were grown to a density of 10^7^ cells·mL^−1^, 30 μL of serial dilutions (OD_600nm_ = 0.5 × 10^0^, 0.5 × 10^−1^, 0.5 × 10^−2^, 0.5 × 10^−3^) were plated on SC-Ura medium containing 0.1 mM hydrogen peroxide or not, and incubated for 72–96 h at 30 °C.

### 4.10. Statistical Analysis

Experiments were performed in completely randomized design. Data were expressed as mean ± standard error. Differences among different treatments were analyzed and compared at the 5% level using SPSS version 7.5 (IBM SPSS, Armonk, NY, USA).

## 5. Conclusions

In this study, three full-length *Trx* cDNAs with different structures and subcellular localization were cloned from banana fruit. Of the three *Trx* genes, the expression of the *MaTrx12* gene was significantly up-regulated by exogenous ethylene treatment that enhanced chilling tolerance, but down-regulated by 1-MCP pretreatment that accelerated chilling injury. Heterologous expression of MaTrx12 in cytoplasmic Trx-deficient *Saccharomyces cerevisiae* strain increased the viability of the strain under H_2_O_2_ treatment. Therefore, it is suggested that MaTrx12 plays an important role in the chilling tolerance of harvested banana fruit, possibly by regulating redox homeostasis.

## Figures and Tables

**Table 1:** Pimers used for the amplification of banana *Trx* genes full cDNA length, construction of eGFP and pFL61 vectors and qPCR.

Name	Nucleotide Sequence (5’-3’)
*Trx*-For1	CGACTTCGCCGCCACNTGGTGYGGNC
*Trx*-For2	CGTGGAGTTCGTCAAGATCGAYGTNGAYGA
*MaTrx6*-5RACE1	CGGAGTACTGGGCGGCCATC
*MaTrx6*-5RACE2	CTCCACGAAGCGGCACGGC
*MaTrx9*-5RACE1	TGTTCCTCGTGGTTTAGTTG
*MaTrx9*-5RACE2	ATCCTCAGCCGACTTGATA
*MaTrx12*-5RACE1	GTTGCTGAGATGTCCCACGA
*MaTrx12*-5RACE2	GCCACAACTATCCTGCCATC
eGFP-*MaTrx6*-Sal I-F	AGTCGACATGGGTGGTTTCTTCTCCAGCC
eGFP-*MaTrx6*-Sal I-R	AGTCGACGGTGTTGATCTGCTCTTGGATCC
eGFP-*MaTrx9*-Mlu I-F	CGACGCGTATGGCGGATGCTTTGGCTC
eGFP-*MaTrx9*-Mlu I-R	CGACGCGTTCTACCGGCTGCGGC
eGFP-*MaTrx12*-Sal I-F	AGTCGACATGGGAAACTGCATAAGAAAGAATGTGAG
eGFP-*MaTrx12*-Sal I-R	AGTCGACTCGTAGCGGGAGAGTAGCATC
*MaTrx6*-F	AGGAGGTGGGCAGAATCGT
*MaTrx6*-R	TGGTGGCGGTAATACAGACAG
*MaTrx9*-F	TCACCTACACAAGAACGCCT
*MaTrx9*-R	CCACATACATAACCAATAAGCAG
*MaTrx12*-F	TCGTGGGACATCTCAGCAAC
*MaTrx12*-R	CTTTCTTCATCGTAGCGGGA
*Actin*-F	TGGTATGGAAGCCGCTGGTA
*Actin*-R	TCTGCTGGAATGTGCTGAGG
pFL61-*MaTrx6*-Not I-F	TTGCGGCCGCATGGGTGGTTTCTTCTCCAGCC
pFL61-*MaTrx6*-Not I-R	TTGCGGCCGCTTAGGTGTTGATCTGCTCCTGGATCC
pFL61-*MaTrx9*-Not I-F	TTGCGGCCGCATGGCGGATGCTTTGGCTC
pFL61-*MaTrx9*-Not I-R	TTGCGGCCGCTCATCTACCGGCTGCGGCAAAAG
pFL61-*MaTrx12*-Not I-F	TTGCGGCCGCATGGGAAACTGCATAAGAAAG
pFL61-*MaTrx12*-Not I-R	TTGCGGCCGCTCATCGTAGCGGGAGAGTAGC
FLDR	CTATTATTTTAGCGTAAAGGATGG
FLGA	CTCTTTTTTACAGATCATCAAGG

## Abbreviations

1-MCP	1-Methylcyclopropene
CaMV	Cauliflower Mosaic Virus
CI	Chilling Injury
GFP	Green Fluorescent Protein
H_2_O_2_	Hydrogen peroxide
iTRAQ	Isobaric Tags for Relative and Absolute Quantitation
ORF	Open Reading Frame
PEG	Polyethylene Glycol
ROS	Reactive Oxygen Species
Trx	Thioredoxin
